# Emendation of the Coccoid Cyanobacterial Genus *Gloeocapsopsis* and Description of the New Species *Gloeocapsopsis diffluens* sp. nov. and *Gloeocapsopsis dulcis* sp. nov. Isolated From the Coastal Range of the Atacama Desert (Chile)

**DOI:** 10.3389/fmicb.2021.671742

**Published:** 2021-07-08

**Authors:** Patrick Jung, Armando Azua-Bustos, Carlos Gonzalez-Silva, Tatiana Mikhailyuk, Daniel Zabicki, Andreas Holzinger, Michael Lakatos, Burkhard Büdel

**Affiliations:** ^1^University of Applied Sciences Kaiserslautern, Pirmasens, Germany; ^2^Centro de Astrobiología (CSIC-INTA), Madrid, Spain; ^3^Instituto de Ciencias Biomédicas, Facultad de Ciencias de la Salud, Universidad Autónoma de Chile, Santiago, Chile; ^4^Facultad de Ciencias, Universidad de Tarapacá, Arica, Chile; ^5^M. G. Kholodny Institute of Botany, National Academy of Sciences of Ukraine, Kyiv, Ukraine; ^6^Institute of Botany, University of Innsbruck, Innsbruck, Austria; ^7^Technical University of Kaiserslautern, Kaiserslautern, Germany

**Keywords:** Chroococcidiopsis, Gloeocapsopsis, Atacama Desert, Chroococcidiopsidales, polyphasic approach

## Abstract

The taxonomy of coccoid cyanobacteria, such as *Chroococcidiopsis*, *Pleurocapsa*, *Chroococcus*, *Gloeothece*, *Gloeocapsa*, *Gloeocapsopsis*, and the related recent genera *Sinocapsa* and *Aliterella*, can easily be intermixed when solely compared on a morphological basis. There is still little support on the taxonomic position of some of the addressed genera, as genetic information is available only for a fraction of species that have been described solely on morphology. Modern polyphasic approaches that combine classic morphological investigations with DNA-based molecular analyses and the evaluation of ecological properties can disentangle these easily confusable unicellular genera. By using such an approach, we present here the formal description of two novel unicellular cyanobacterial species that inhabit the Coastal Range of the Atacama Desert, *Gloeocapsopsis dulcis* (first reported as *Gloeocapsopsis* AAB1) and *Gloeocapsopsis diffluens*. Both species could be clearly separated from previously reported species by 16S rRNA and 16S–23S ITS gene sequencing, the resulting secondary structures, p-distance analyses of the 16S–23S ITS, and morphology. For avoiding further confusions emendation of the genus *Gloeocapsopsis* as well as epitypification of the type species *Gloeocapsopsis crepidinum* based on the strain LEGE06123 were conducted.

## Introduction

The morphological characteristics of unicellular thylakoid-lacking cyanobacteria, such as *Gloeobacter violaceus*, *Gloeobacter kilaueensis*, *Aurora vandensis* (Grettenberger et al., 2020), and the thylakoid-bearing genus *Chroococcidiopsis* are thought to be similar to Proterozoic microfossils and have been proposed as some of the most ancient cyanobacteria on Earth (Schirrmeister et al., 2013). From the Proterozoic on, cyanobacteria, and especially unicellular species, occupied many extreme environments on Earth. *Chroococcidiopsis* species have been found in Antarctica (Das and Singh, 2017), hot deserts (Büdel, 1999), freshwater, marine and hypersaline environments (Dor et al., 1991), and living in symbiosis with lichens (Büdel and Henssen, 1983; Büdel et al., 2000). Given their tolerance to a wide range of environmental stresses (high UV radiation, extreme desiccation, and high salinity), members of this genus have been proposed as model organisms for the colonization of Mars (Puente-Sánchez et al., 2018a; Billi et al., 2019; de Vera et al., 2019; Mosca et al., 2019).

In addition to Chroococcidiopsis, other unicellular genera from extreme environments, such as *Gloeocapsopsis*, are gaining attention; *Gloeocapsopsis* sp. AAB1 (Azua-Bustos et al., 2014; in the present study *Gloeocapsopsis dulcis* sp. nov.,) was first isolated from hypolithic biofilms under quartz stones of the Coastal Range of the Atacama Desert and showed extreme desiccation tolerance (Azua-Bustos et al., 2014). In 2018, the ∼5.4-Mb sized genome of this strain was published giving insights into the unique genetic potential related to the biosynthesis and regulation of compatible solutes and polysaccharides (Puente-Sánchez et al., 2018b). Despite many reports on this genus, the taxonomic placement of some genera of the Chroococcidiopsidales is still unclear as the current classification has encountered severe problems. With the recent report of the genus *Sinocapsa* (Wang et al., 2019), the Chroococcidiopsidales now comprise four separate coccoid cyanobacterial clusters at the vicinity of the Nostocales, apart from coccoid cyanobacterial orders, such as the Chroococcales, Pleurocapsales, and Synechococcales (Wang et al., 2019). These four main clusters were established based on the genus *Sinocapsa* in cluster A, the genus *Aliterella* of the family Aliterellaceae in cluster B, *Chroococcidiopsis sensu stricto* of Chroococcidiopsidaceae in cluster C, and the genera *Chroogloeocystis*, *Gloeocapsa*, and *Gloeocapsopsis* in cluster D (according to Wang et al., 2019). However, the Chroococcidiopsidales appear to be a polyphyletic group since the three genera of Oscillatoriales *Cephalothrix*, *Aerosakkonema*, and *Microseira* are phylogenetically mixed into the Chroococcidiopsidales. They are close to *Chroococcidiopsis* but distant to the other genera of the Chroococcidiopsidales (Wang et al., 2019).

The type species of *Gloeocapsopsis*, *G. crepidinum* (Komárek, 1993), was described in 1854 by Thuret as *Protococcus crepidinum* based on natural material. Therefore, it does not have an authentic strain, and material for genetic analyses is unavailable. For this reason, we attempted an epitypification of *G. crepidinum* with the strain *G. crepidinum* LEGE06123 (Ramos et al., 2010) as a valid reference point for the genus *Gloeocapsopsis*. This enabled us to describe the two novel strains *G. dulcis* and *G. diffluens* found in the Coastal Range of the Atacama as two new species, and in this way strengthen the taxonomy around the Chroococcidiopsidales. Further, this article summarizes detailed information on all other proposed *Gloeocapsopsis* species described solely on morphological features, which can act as a baseline work for the description of members of the genus following the standards of the polyphasic approach.

## Materials and Methods

### Sampling Locations, Isolation Procedures, and Strain Maintenance

**Figure 1:**
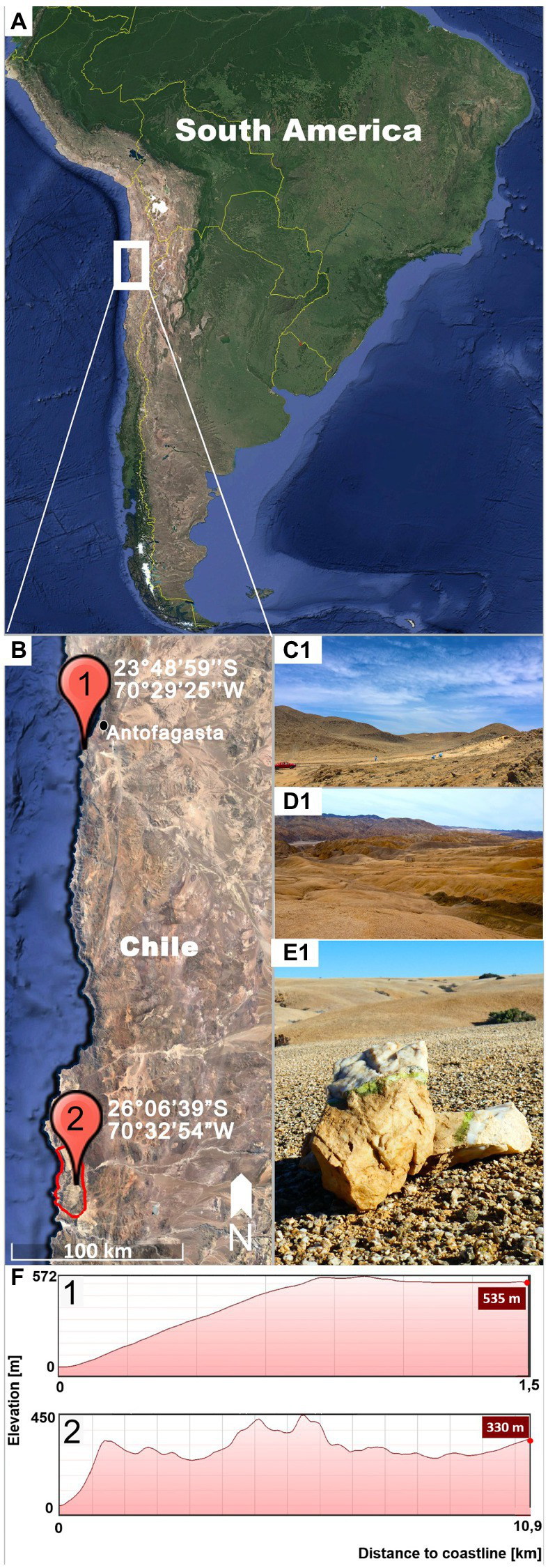
Sampling locations. **(A)** shows on overview of South American indicating the location of the inspected region (white square). **(B)** Detail of the Coastal Range of the Atacama Desert showing the sampling sites of *Gloeocapsopsis dulcis* sp. nov. (marked with **1**) and *G. diffluens* sp. nov. (marked with **2**) in the National Park Pan de Azúcar (red circle). **(C)** Landscape of the sampling site **(1)** of *G. dulcis* sp. nov. **(D)** Landscape of the sampling site **(2)** of *G. diffluens* sp. nov. **(E)** Quartz stone showing green hypolithic biofilm from which *G. diffluens* sp. nov. was isolated. **(F)** Topography of both sampling sites **(1,2)** representing elevation and distance from coastline.

*Gloeocapsopsis* sp. AAB1 was first found by Armando Azua-Bustos and Carlos Gonzalez-Silva in a hypolithic microbial community under a quartz rock in the Coastal Range of the Atacama Desert (Azua-Bustos et al., 2014; Figures 1A,B1,C), and then isolated by Jorge Zúñiga. This site is located 22 km south of the city of Antofagasta (23°48′59″S, 70°29′25″W, 535 m a.s.l.), in the eastern slope of the hills of the Coastal Range and about 1.5 km from the Pacific Ocean (Figure 1F1).

The strain *Gloeocapsopsis* sp. PJ S16 was isolated from a hypolithic biofilm found under quartz stones at the Pan de Azúcar National Park, located at the Coastal Range of the Atacama Desert (Figures 1A,B2,D,E), during a previous study as described therein (Jung et al., 2019). The area is 10 km off the coastline (26°06′39″S, 70°32′54″W, 330 m a.s.l.; Figure 1F2) with sparse vegetation mainly of the endemic cactus species *Copiapoa cinerea*ssp. *columna-alba*, where fog and dew are the main sources of water (Lehnert et al., 2018). In this site a recently reported grit crust (a transitional stage between a biocrust and a saxicolous community made of lichens, fungi, and algae growing attached to the granitoid substrate) covers the ground forming blackish patterns (Jung et al., 2020a). From this area, only a few cyanobacteria, such as *Kastovskya adunca*, *Pleurocapsa* sp., *Chroococcidiopsis* sp., *Pseudophormidium* sp., *Nostoc* sp., and the recently described *Aliterella chasmolithica* (Jung et al., 2020b), have been reported to appear in the aridisol as well as hypolithic and chasmoendolithic biofilms attached to exposed granite and quartz pebbles (Jung et al., 2019).

The type strain *G. crepidinum* LEGE06123 was included in the descriptions given here by means of an epitypification and obtained from the Culture Collection of Algae and Protozoa (CCAP) as strain CCAP 1425/1 submitted by Ramos et al. (2010). Originally, the strain was isolated from the surface of green seaweeds and dark mats in shallow puddles that retain seawater during low tide at the Algarve coast, Portugal in 2008 (Ramos et al., 2010).

The strains were maintained on solidified and liquid BG11 medium (Stanier et al., 1971) at 20°C and 25 μmol photons m^−2^ s^−1^ (Osram Lumilux Cool White lamps L36W/840) in a light/dark cycle of 12:12 h L:D.

### Morphological Characterization

The morphology of the three *Gloeocapsopsis* isolates was inspected weekly over the course of several months by bright field microscopy using a Leica DM1000 LED (Leica Microsystems GmbH, Wetzlar, Germany), equipped with a 100× magnification and oil immersion coupled with a Leica MC170 HD digital microscope camera and the software LAS V4.3 (Leica Microsystems GmbH, Wetzlar, Germany). This was carried out on cultures on BG11 agar plates as well as liquid BG11 medium. Fifty images were taken from each strain. Length and width were measured in 300 cells with ImageJ 1.47v. Digital drawings were made with an Ugee M708 touchpad and Adobe Photoshop CS6 based on microscopic images.

### Transmission Electron Microscopy

High-pressure freeze (HPF) fixation and freeze substitution (FS) protocols were applied as described (Aichinger and Lütz-Meindl, 2005) for *Gloeocapsopsis* sp. PJ S16. Two-month-old cultures were fixed with a LEICA EMPACT high-pressure freezer and freeze substituted in a Leica EM AFS FS (Leica Microsystems GmbH, Vienna, Austria) apparatus, in 2% OsO4 and 0.05% uranyl acetate in acetone at −80°C for 60 h, the temperature was raised to −30°C within 5 h (10°C/h), maintained at −30°C for 4 h, and then temperature was raised to 20°C within 20 h (2.5°C/h). Samples were embedded in an agar low viscosity resin kit (Agar Scientific, Essex, United Kingdom). Ultrathin sections were prepared with a Reichert Ultracut S (Leica AG, Vienna, Austria) and stained with 2% uranyl acetate and lead citrate. Samples were then examined with a Zeiss Libra 120 transmission electron microscope at 80 kV and photographed with a 2 × 2 k digital high-speed camera (Tröndle, Moorenweis, Germany) under control of ImageSP software (Tröndle, Moorenweis, Germany).

In the case of *Gloeocapsopsis* sp. AAB1 transmission electron microscopy was performed as already described in Azua-Bustos et al. (2014).

### DNA Extraction, Amplification, and Sequencing

Genomic DNA of *Gloeocapsopsis* sp. PJ S16 was extracted from unialgal cultures as described by Jung et al. (2020b). Nucleotide sequences of the 16S rRNA gene together with the 16S–23S ITS region (1,700–2,300 bases) were amplified as described by Marin et al. (2005) using the primers SSU-4-forw and ptLSU C-D-rev. The PCR products were cleaned using the NucleoSpin Gel and PCR Clean-up Kit (Macherey-Nagel GmbH and Co. KG) following the DNA and PCR clean-up protocol. The cleaned PCR products were sent to Seq-It GmbH and Co. KG (Kaiserslautern, Germany) for Sanger sequencing with the primers SSU-4-for, Wil 6, Wil 12, Wil 14, Wil 5, Wil 9, Wil 16, and ptLSU C-D-rev (Wilmotte et al., 1993; Marin et al., 2005; Mikhailyuk et al., 2016). Sequences were then assembled and manually edited by removing ambiguous base pairs in consensus with the electropherograms of the single sequences where appropriate using the software Mega X version 10.0.5 (Kumar et al., 2018). All sequences were submitted at NCBI GenBank as stated in the species description.

### Molecular Characterization

*Gloeocapsopsis* sequences were compared against the NCBI GenBank database^1^ in order to find the most similar sequences, which were subsequently incorporated into the alignment using *Gloeobacter* as an outgroup. In addition, 16S sequences of representatives comprising all cyanobacterial orders were added. The alignment was prepared applying for the Muscle alignment program in Mega X. Finally, 121 nucleotide sequences were used for the phylogenetic comparison including 1,503 bp of the 16S rRNA gene of most species here studied. Ambiguous regions within the alignment were adjusted or removed manually allowing smaller final blocks and gap positions within the final blocks. The evolutionary model that was best suited to the used database was selected on the basis of the lowest Akaike’s Information Criterion value and calculated in Mega X which turned out to be Kimura 2 + G + I model. This model was used to construct the phylogenetic tree with Mega X. The maximum likelihood method (ML) with 1,000 bootstrap replications was calculated in Mega X.

Bayesian phylogenetic analyses were carried out with Mr. Bayes 3.2.1 (Ronquist and Huelsenbeck, 2003) using an evolutionary model GTR + G + I. For the Bayesian analysis, two runs of the four Markov Chain Monte Carlo were made simultaneously, with the trees taken every 500 generations. Split frequencies between runs were below 0.01 at the end of the calculations. The trees selected before the likelihood rate reached saturation were subsequently rejected.

In addition, percent dissimilarity among aligned 16S–23S ITS regions were displayed by calculating 100× uncorrected p-distance in Mega X (Erwin and Thacker, 2008; González-Resendiz et al., 2019; Shalygin et al., 2019). This allowed to have a discontinuity between percent dissimilarity of populations in the same species (average ~1.0% or less, all pair-wise comparisons <3% dissimilarity) and populations representing separate species (>7% dissimilarity; González-Resendiz et al., 2019). When differences are between 3 and 7%, the cutoff is not clear and a decision can be based on the other criteria, such as 16S phylogeny or morphology. Models of the secondary structure of 16S–23S ITS region of both new *Gloeocapsopsis* species in comparison with *G. crepidinum* were built according to the models proposed in Wang et al. (2019). Helices were folded with the online software Mfold (Zuker, 2003) and visualized in the online tool PseudoViewer (Byun and Han, 2009).

### Holotype Preparation

Species were described following the rules and requirements of the International Code of Nomenclature for algae, fungi, and plants (Turland et al., 2017). Furthermore, young (3 weeks old) cultures of *Gloeocapsopsis* sp. PJ S15, *Gloeocapsopsis* sp. AAB1, and *G. crepidinum* LEGE06123 were preserved in 4% formaldehyde, in 15 ml glass bottles. Preserved material was then deposited in the Herbarium Hamburgense, Hamburg, Germany (HBG-024928, 29, 31). In addition, TEM samples of *Gloeocapsopsis* sp. PJ S15 fixed and embedded in resin are available at the Department of Botany, University of Innsbruck, Austria.

## Results

**Figure 2:**
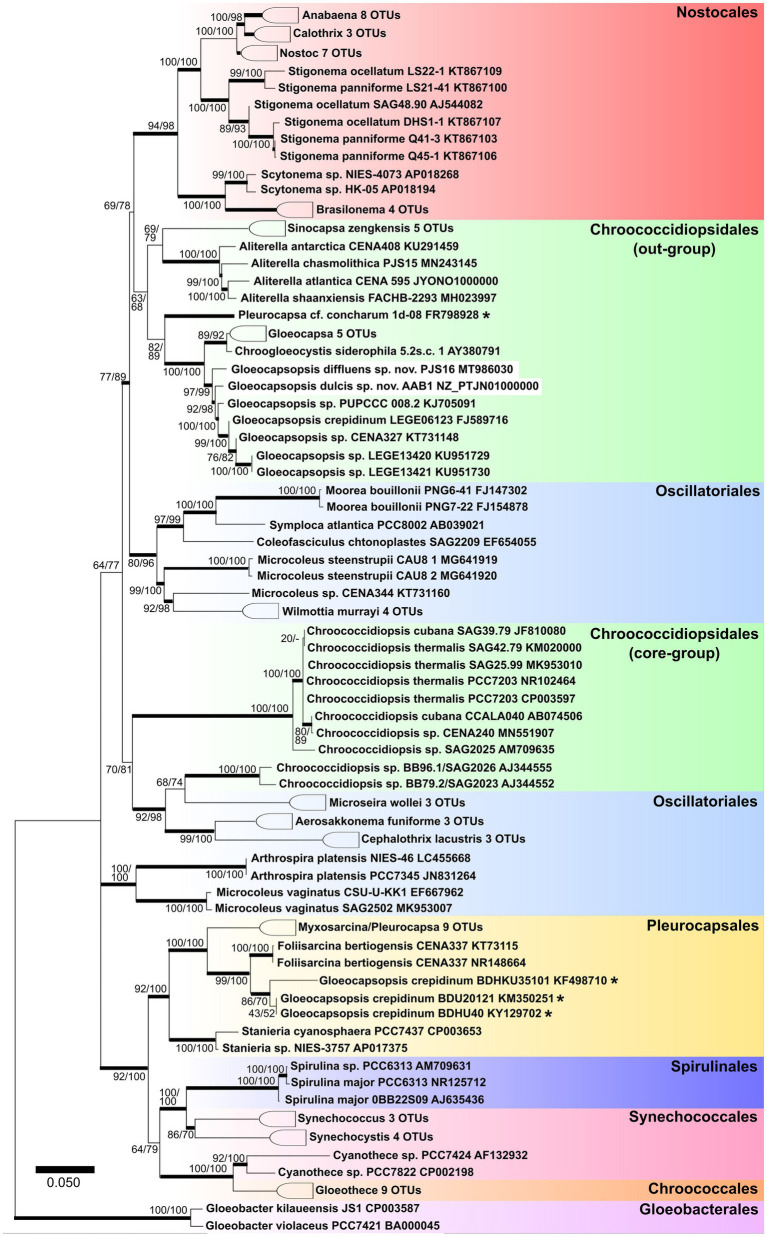
Maximum likelihood tree obtained from 121 aligned 16S rRNA gene sequences of all known orders of cyanobacteria. Numbers on the nodes represent ML and Bayesian bootstrap values, respectively, (1,000 replicates). Bold horizontal lines show lineages supported by at least 75% of bootstrap values. Asterisks identify sequences with ambiguous taxonomic assignments.

Both *Gloeocapsopsis* strains were found to be unique based on their ecology, morphology, ultrastructure, distribution, phylogeny (Figure 2), p-distance analysis of the 16S–23S ITS, and 16S rRNA secondary structures. Because the combination of diacritical features associated with these species did not correspond with any described species within the genus *Gloeocapsopsis*, we hereby named these two novel strains *G. dulcis* sp. nov. AAB1 and *G. diffluens* sp. nov. PJ S15 along with the emendation of *G. crepidinum*.

### Proposed Taxonomic Treatment: *Gloeocapsopsis* Geitler ex Komárek emend. P. Jung et B. Buedel

#### Original Descriptions

Geitler (1925) Beih. Bot. Centralbl. 41. Abt. II: 229. sine diagn.: Komárek (1993) Bull. Natl. Sci. Mus. Tokyo, Ser. B (Bot.), 19: 24.

#### Emended Diagnosis

Forms mat-like structures in liquid cultures that easily disintegrate into single, rounded aggregates of one millimeter. Rarely solitary cells, mostly micro- or macroscopic, irregular, formless, or granular colonies, composed of densely, irregularly aggregated cells or their small groups, surrounded by mucilaginous envelopes. Cells subspherical, more or less irregular rounded in outline, sometimes slightly elongate (never spherical or ellipsoidal) enveloped by thin, narrow, and clearly delimited, sometimes diffluent toward outer periphery, sometimes feebly lamellate, and sometimes colored sheaths, usually following the cell outline. Tetrads sometimes present; nanospores were not observed. Cell division is irregularly in various planes in successive generations. Reproduction by liberation of divided end ensheathed cells from ruptured mother sheaths. The occasional presence of enlarged resting cells with thick, firm, and usually intensely colored envelopes can be observed.

#### Type Species

**Figure 3:**
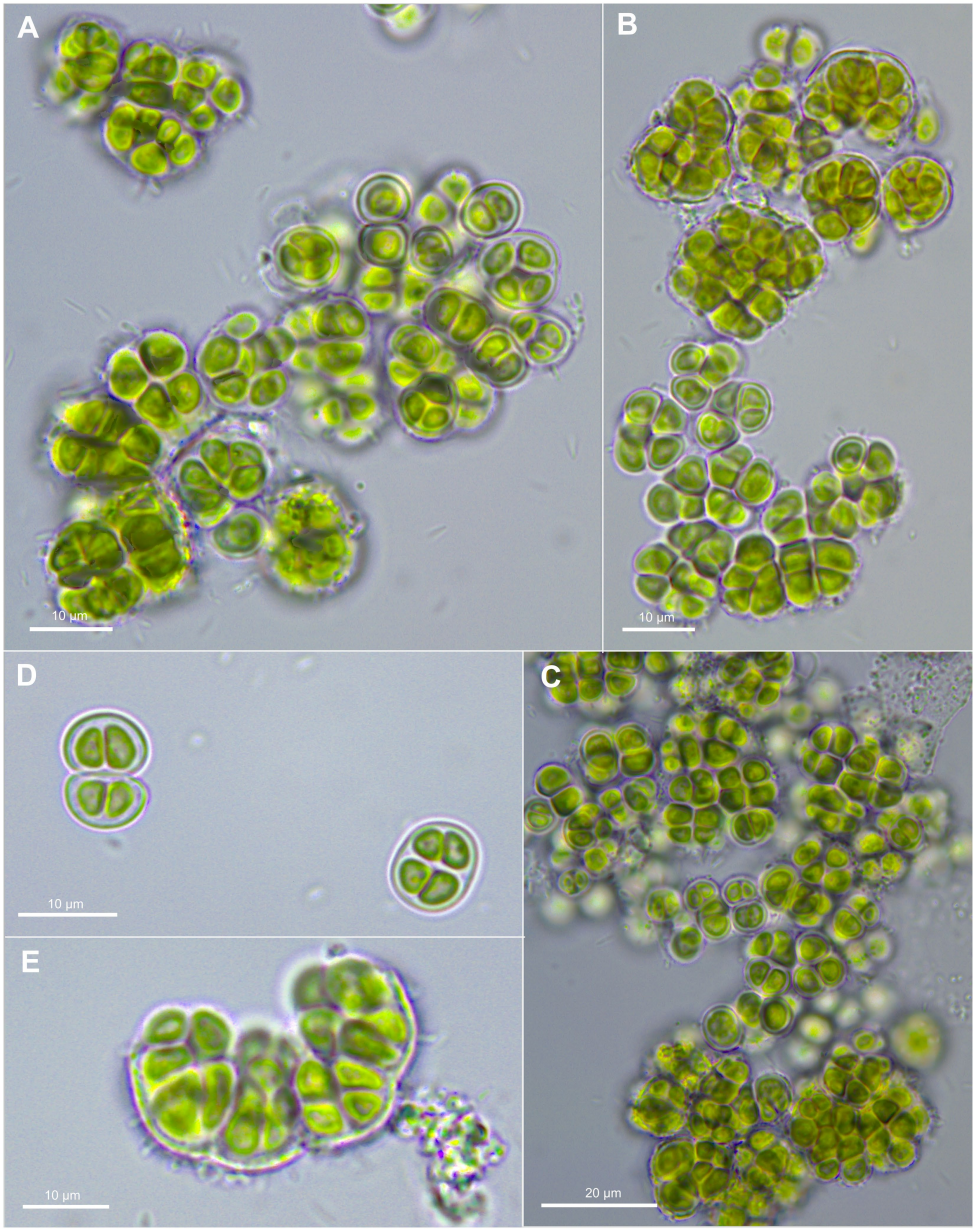
Morphological characteristics of *G. crepidinum* strain LEGE06123. **(A-C)** show single cells and macrocolonies with thick, limited, and hyaline sheaths. **(D)** shows a pair of double cells (upper left corner), each surrounded by a sheath and tetrad of cells (lower right corner). **(E)** shows the rupture of macrocolony.

*Gloeocapsopsis crepidinum* (Thuret) Geitler ex Komárek emend. P. Jung, M. Lakatos et B. Buedel; Figure 3.

#### Comments

*Gloeocapsopsis* differs from other coccoid cyanobacterial genera by their dense but not squeezed aggregation of cells that have an irregularly rounded but never spherical or elongated form surrounded by a firm envelope.

### *Gloeocapsopsis crepidinum* (Thuret) Geitler ex Komárek emend. P. Jung, M. Lakatos et B. Buedel

#### Basionym

*Protococcus crepidinum* Thuret (1854) Mém. Soc. Imp. Sci. Nat. Cherbourg 2: 388 (Figure 3).

#### Synonyms

*Pleurococcus crepidinum* (Thuret) Rabenhorst, *Gloeocapsa crepidinum* (Thuret) Thuret, *Chroococcus crepidinum* (Thuret) Hansgirg, and *Pleurocapsa crepidinum* (Thuret) Ercegović.

#### Emended Diagnosis

Cells aggregating, up to macroscopic clumps or mats of a few millimeters, gelatinous, irregular, olive-green to blue-green when young. Cells irregular-spherical, (2.5) 4–8 μm in diameter, with thin, colorless, limited, and non-lamellate sheath; outer envelopes hyaline, diffluent. Cell content is vivid olive green or slightly blue-grayish, usually homogeneous, not granulated. Tetrad formation present and nanospores absent; Reproduction by liberation of divided end ensheathed cells from ruptured mother sheaths. The parietal thylakoid position is visible in light microscopy.

#### Type Locality

France: Manche, near Cherbourg.

#### Epitype (Designated Here)

The preserved specimen of material of the strain *G. crepidinum* LEGE06123 (CCAP 1425/1) is available *via* the Herbarium Hamburgense, Hamburg, Germany (HBG-024929).

#### Epitype Strain

*Gloeocapsopsis crepidinum* LEGE06123 (CCAP 1425/1) was isolated from the intertidal diazotrophic mat at Luz, a rocky beach in southern Portugal.

#### Comments

The epitype strain completely corresponds to the diagnosis of *G. crepidinum* (Thuret, 1854; Komárek, 1993). The species diagnosis was supplemented by the details of reproduction style, presence of tetrads, absence of nanospores, and description of form and color of macroscopic aggregates. The epitype strain was isolated from the intertidal diazotrophic mat at Luz (Portugal). This habitat is ecologically comparable to the characteristic of the species as halophilic, typical for the littoral and sprayed supralittoral of seas and inland saline lakes and swamps (Komárek and Anagnostidis, 1998). It is also similar to the type locality and is a part of the same geographical region (Europe).

### *Gloeocapsopsis dulcis* sp. nov. A. Azua-Bustos et P. Jung

#### Description

**Figure 4:**
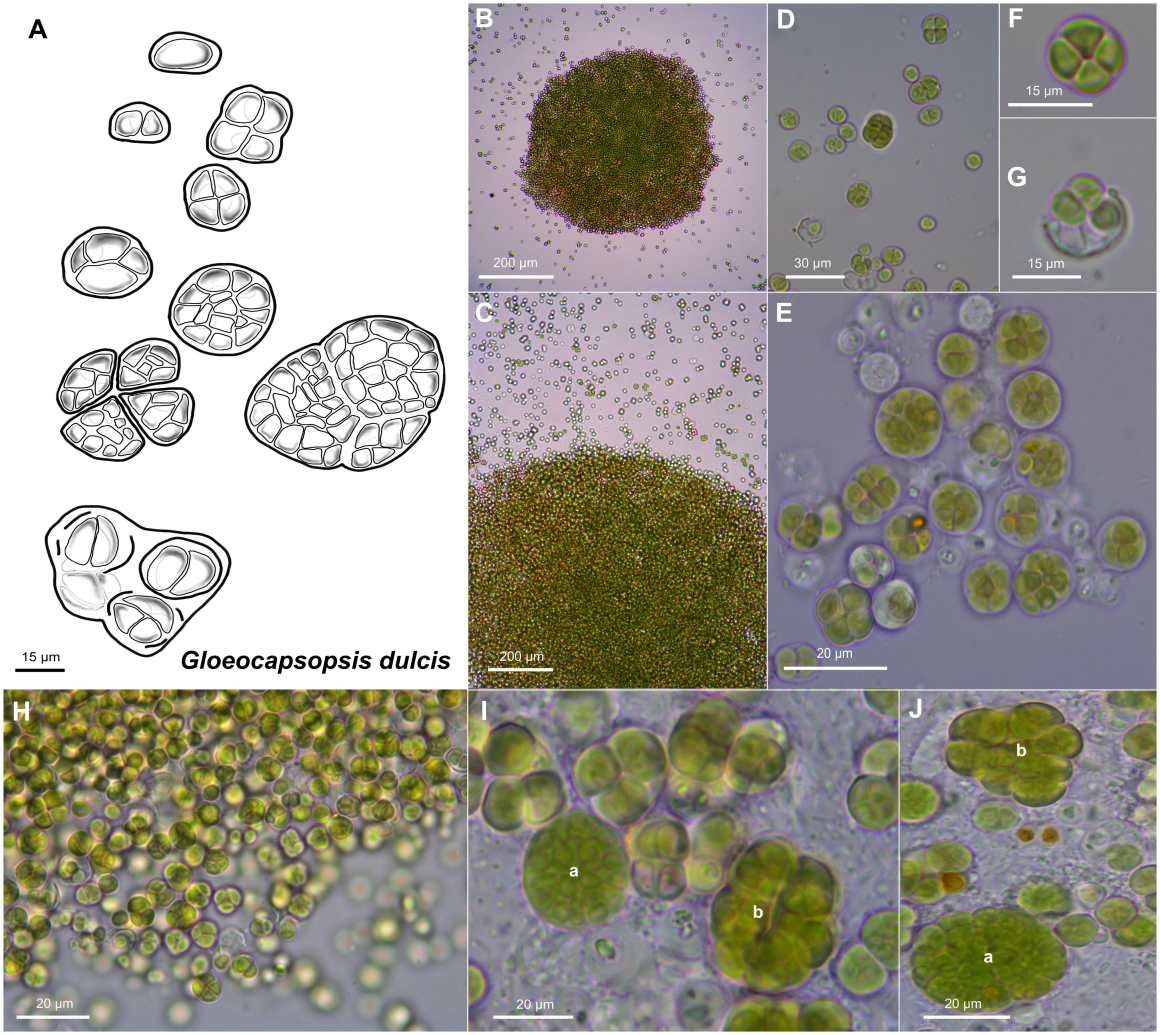
Morphological characteristics of *G. dulcis* sp. nov. strain AAB1. **(A)** Scheme summarizing the life cycle of *G. dulcis*. **(B,C)** show microcolonies releasing single cells and small cell aggregates. **(D,E)** depict various types of aggregates. **(F)** shows a tetrad of cells. **(G)** shows single cells been released from a previously shared sheath. **(H)** depicts the aspect of a young culture. **(I,J)** show type *a* colonies made of densely packed cells surrounded by a firm sheath, and type *b* colonies made of cell packages surrounded by firm sheath mimicking the form of the cells.

**Figure 5:**
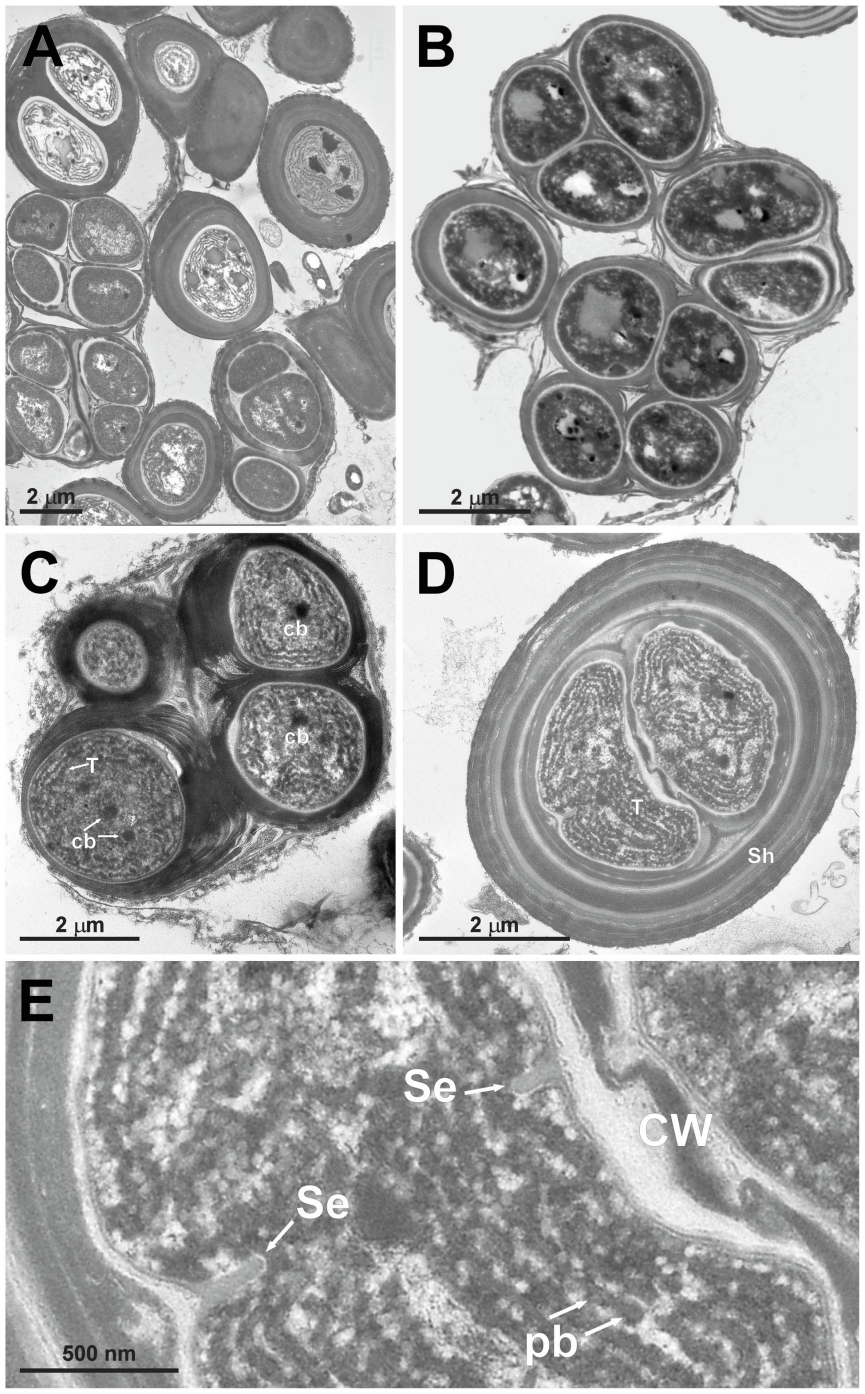
TEM micrographs of *Gloeocapsopsis dulcis* sp. nov. strain AAB1. **(A,B)** show the diffluent shape of colonies. **(C)** depicts a tetrad of cells with carboxysomes (cb) and parietal thylakoid membranes (T). **(D)** shows a cell embedded in multiple layers of dense extracellular polymeric substances and intracellular sheaths (Sh). **(E)** shows a detail of **(D)**, depicting the cortical area with thylakoids, phycobilisomes (pb), and the incipient formation of a septum (Se). CW, cell wall.

Unicellular, spherical cells, 2.4(±0.2) μm × 2.2(±0.3) μm and 3.3(±0.4) µm in diameter, often angularly clinched, and heterogeneous in size during colony development (Figures 2, 4, and 5; Supplementary Tables 1 and 2). Cell content is homogeneous, non-granulated, green to olive green, and never blue, with parietal thylakoid membranes and thylakoid-free nucleoplasm in the center. The sheath of single cells is always colorless, slightly lamellate, and limited with up to 1 μm thickness. Binary fission of mother cells leads to division in different directions, without nanocytes. Cells often agglomerated into irregular packets of two or more cells, often forming tetrads, surrounded by a common firm, limited, sometimes slightly lamellate, and colorless sheath. Older colonies can be observed as two types: (1) packages containing small, geometrically clinched cells embedded together in a common colorless, limited, and firm sheath forming a rounded pattern; (2) tetrad-like packages with cells unitedly clinched in packages of only a few cells that are agglomerated densely with other packages of about the same size, held together by a colorless sheath that firmly mimics the shape of the cell packages.

#### Transmission Electron Microscopy

*Gloeocapsopsis dulcis* sp. nov. AAB1: Figures 5A,B show the accumulation of individual cell colonies, with smaller packages of tetrads in Figure 5C. Cell divisions were observable by the formation of a septum (Figure 5E), giving rise to new colonies. In most cases, daughter cells remained together inside the sheath of the mother cell (Figures 5B–D). Thylakoids showed a small folded parietal structure with dense cyanophycin granules, and filling at least two-thirds of the cell interior with a thylakoid-free nucleoplasma in its center (Figure 5D). Carboxysomes can also be clearly seen (Figure 5C). Thylakoids with visible phycobilisomes (Figure 5E) were pushed inward during the formation of the septum (Figure 5E). Each cell in the colony is enclosed by a cell wall (Figure 5C), and the colony is embedded in an up to 1 μm thick, multi-layered sheath with varying electron densities (Figures 5D,E).

#### Habitat

Hypolithic biofilm under quartz stones of the Coastal Range of the Atacama Desert.

#### Etymology

‘*dulcis*’ – ‘sweet’, due to the high contents of trehalose and sucrose that the species accumulates when confronting desiccation (Azua-Bustos et al., 2014).

#### Type Locality

CHILE – Coastal Range of the Atacama Desert, 22 km south of the city Antofagasta (23°48′59″S; 70°29′25″W, 537 m a.s.l.), and eastern hill slope 1.5 km from the Pacific Ocean. First collected on 10.09.2005 by Armando Azua-Bustos and Carlos Gonzalez-Silva, and then isolated by Jorge Zúñiga and Armando Azua-Bustos in 2011.

#### Holotype

The preserved holotype specimen is available *via* the Herbarium Hamburgense, Hamburg, Germany (HBG-024931).

#### Reference Strain

The reference strain *G. dulcis* sp. nov. AAB1 is available at the culture collection DSMZ Braunschweig (DSM 112007).

#### Differentiation Against Other Species

*Gloeocapsopsis dulcis* sp. nov. differs from other *Gloeocapsopsis* species by having the smallest cells of all described species within the genus, and by the two abovementioned types of cell packages that are formed after cell division. It differs from *G. diffluens* sp. nov. PJ S16 by a more prominently laminated sheath in ultrastructure.

#### Phylogenetic Relations and Secondary Structure of the 16S–23S ITS Sequence

**Figure 6:**
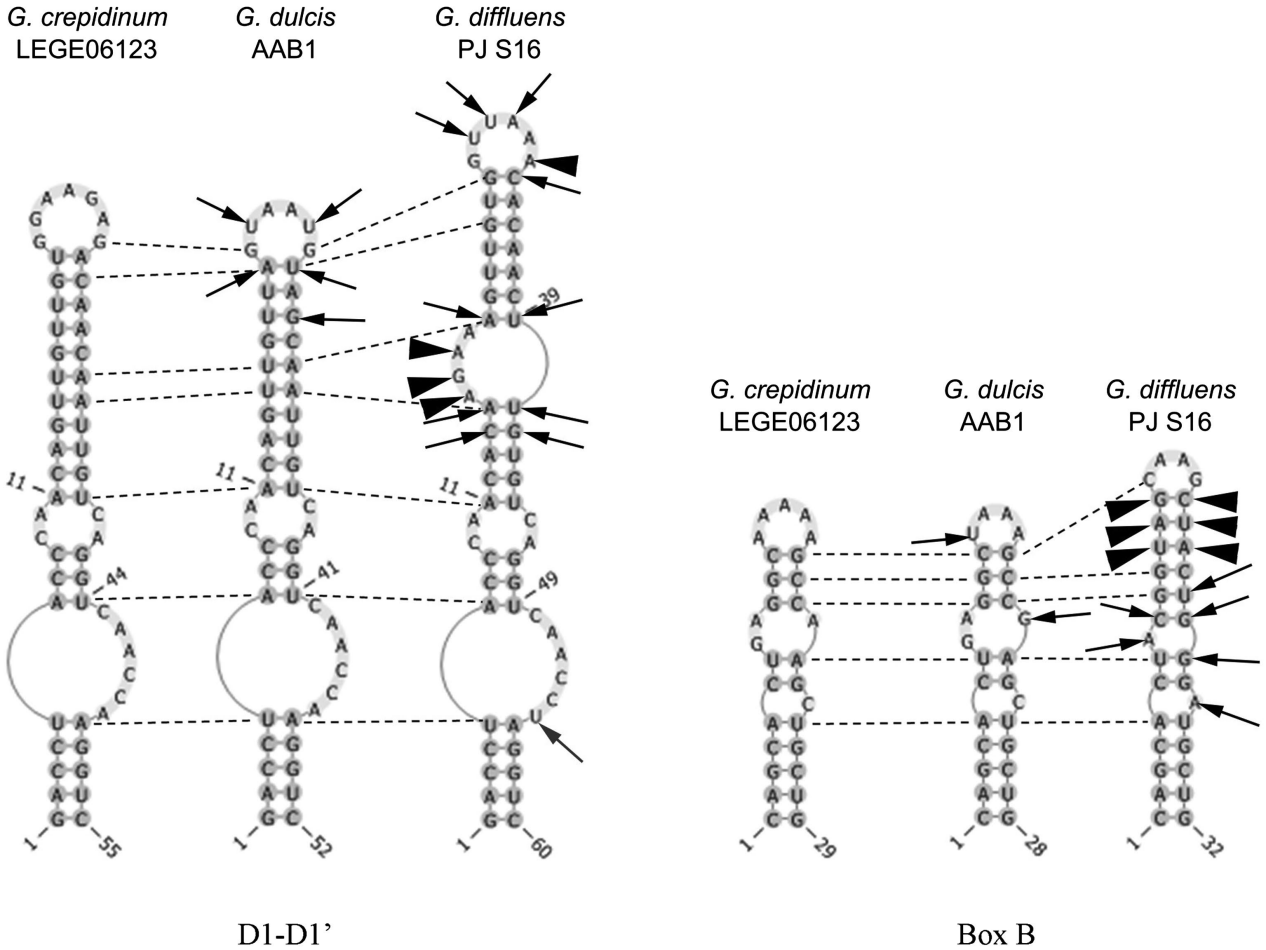
Secondary structure of the main informative helices of the 16-23S ITS region of the inspected species. Differences between strains are presented in comparison with *Gloeocapsopsis crepidinum* strain LEGE 06123. Arrows show variable bases, arrowheads show insertions/deletions of base pairs, and dotted lines show homological base pairs among species.

The sister clade of *Gloeocapsopsis* is *Gloeocapsa*. Based on the 16S rRNA gene sequencing, *G. dulcis* sp. nov. AAB1 is closely related to *G. crepidinum* LEGE 06123 (98.77%), *G. diffluens* sp. nov. PJ S16 (98.29%), *Gloeocapsopsis* sp. CENA 327 (98.23%), and an uncultured clone GI8-sp-D02 (97.45%; accession number GQ129887). The p-distance comparison of the ITS region shows 15.6% dissimilarity with *G. crepidinum* LEGE 06123 and 20.7% with *G. diffluens* sp. nov. PJ S16. Secondary structure of the main informative helices of 16S–23S ITS sequences shows only a few differences in the D1-D1′ region between *G. dulcis* sp. nov. AAB1 and *G. crepidinum*, but some insertions and deletions occurred compared to *G. diffluens* sp. nov. PJ S16 that resulted in the significantly different folding structure of this region (Figure 6). Despite on two positions within Box B, the secondary structure of *G. dulcis* sp. nov. AAB1 and *G. crepidinum* was identical, with great differences compared to *G. diffluens* sp. nov. PJ S16.

### *Gloeocapsopsis diffluens* sp. nov. P. Jung et. B. Büdel

#### Description

**Figure 7:**
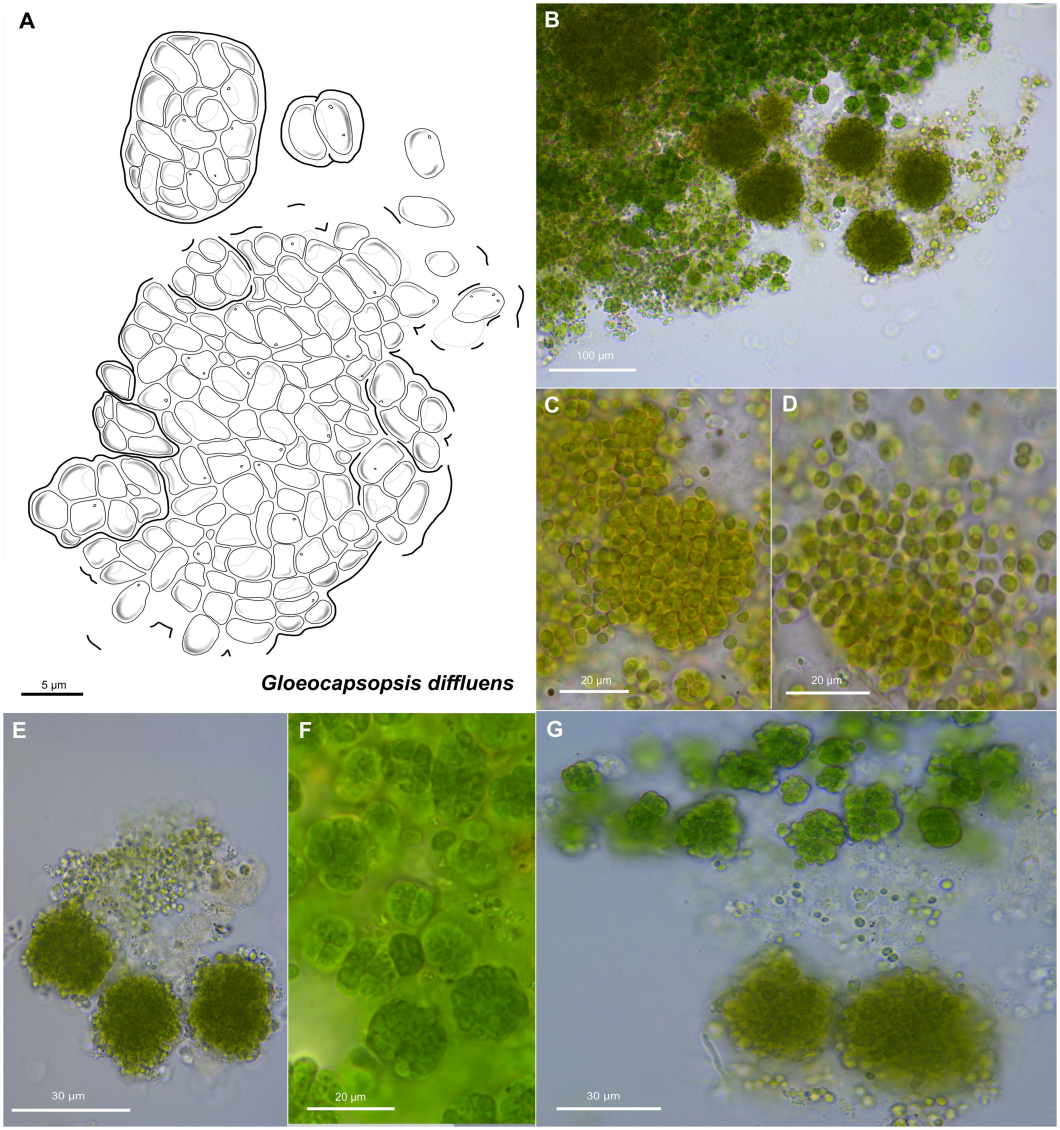
Morphological characteristics of *G. diffluens* sp. nov. strain PJ S16. **(A)** Scheme summarizing the life cycle of *G. diffluens*. **(B)** Overview showing older brownish macrocolonies surrounded by young greenish colonies, made of smaller cell aggregates and single cells. **(C)** *Coleochaete*-like growth of older colonies. **(D,E)** show older colonies with diffluent and interrupted sheaths releasing unsheathed single cells. **(F)** shows various aggregates in a young culture. **(G)** depicts older brownish macrocolonies surrounded by young greenish colonies made of smaller cell aggregates and single cells.

**Figure 8:**
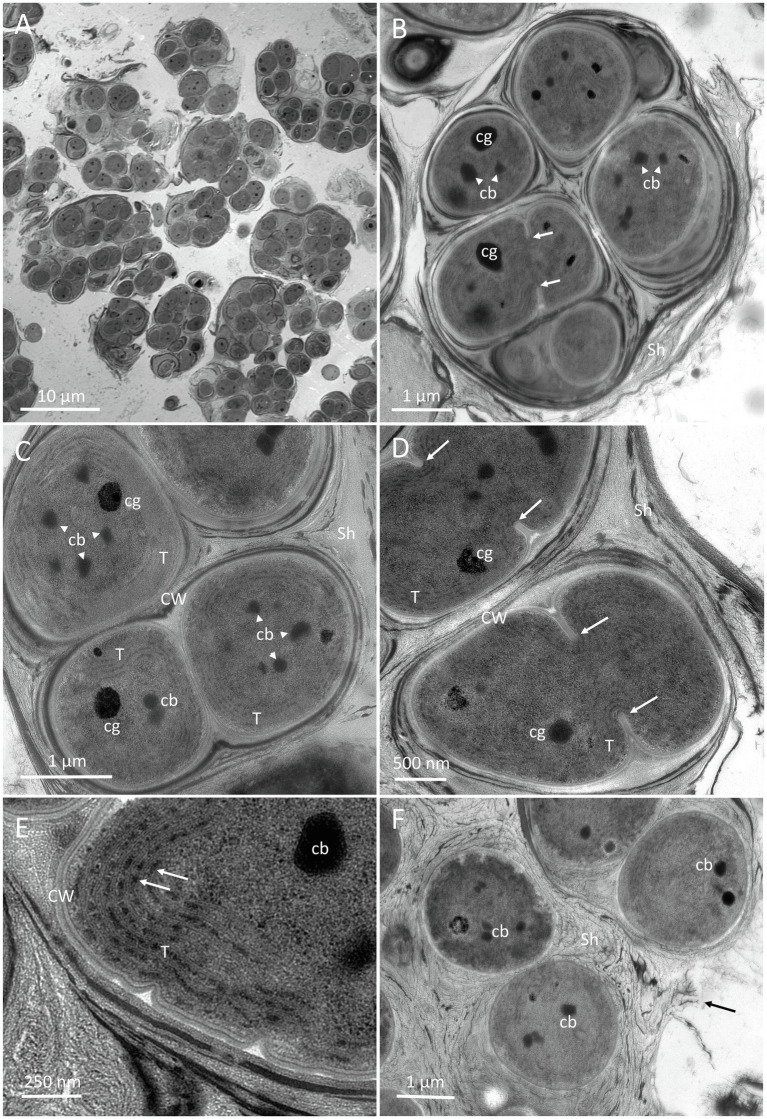
TEM micrographs of *G. diffluens* sp. nov. strain PJ S16. **(A)** depicts the diffluent shape of cell colonies. **(B)** shows a colony of cells, with one of them (arrows), along carboxysomes and lipid bodies in the central cytoplasm. **(C)** Cell colony with one freshly divided cell still in mother cell wall, thylakoids parietal, carboxysomes, and electron dense bodies. **(D)** depicts two dividing cells (arrows pointing to invaginations forming the septum). **(E)** Details of the cortical area, with thylakoid membranes and phycobilisomes (arrows). **(F)** shows cells embedded in a loose extracellular polymeric substances (EPS), which appears to disintegrate. Abbreviations: cb, carboxysome; CW, cell wall; Sh, sheath made of extracellular EPS; and cg, cyanophycin granules.

Unicellular, spherical cells, 3.2(±0.2) μm × 2.4(±0.2) μm, 4.0(±0.3) μm in diameter, and more or less uniform throughout colony development (Figures 2, 7, 8; Supplementary Tables 1 and 2). Cell content is homogeneous, non-granulated, olive green, and sometimes bluish, with parietal thylakoid membranes and thylakoid-free nucleoplasma in its center. Single cells always show colorless, non-lamellate, and limited sheaths of up to 1 μm thickness. Binary fission of mother cells leads to division in different planes, without nanocytes. Cells often agglomerated into irregular packets of many cells, rarely in tetrads, surrounded by a common firm, limited, non-lamellate, and colorless sheath resulting in a rounded shape. Sheaths of colonies become highly diffluent in larger colonies, mimicking the shape of the cells. Older colonies are larger, rounded, and slightly flattened forming a *Coleochaete*-like parenchymatous pattern, with hundreds of cells embedded in a common, colorless, and non-lamellate sheath that becomes diffluent and often unlimited, where unsheathed single cells are released from the periphery.

#### Transmission Electron Microscopy

An overview of two-month-old cultures is shown in Figure 8A, where the loose accumulation of individual cell colonies is visible. Cells were usually arranged in small colonies with the irregular arrangement, surrounded by an irregular sheath with varying electron density. Cell divisions were observable by the formation of a septum (Figures 8B,D, arrows), which gave rise to the formation of colonies. Recently divided cells stayed in the sheath of the thin mother cell (Figure 8C). The central nucleoplasm was free of thylakoids and contained medium electron-dense carboxysomes, clearly detected by their polygonal shape (Figures 8B,C). The electron density of the carboxysomes varied (Figure 8E). Other electron-dense granules were determined as cyanophycin granules due to their round appearance and their radiating pattern (Figure 8C). Thylakoids showed a parietal arrangement (Figure 8C), and during septum formation were pushed inward (Figure 8D). At a higher magnification, distinct electron-dense phycobilisomes were clearly visible on thylakoid membranes (Figure 8E). Cells in colonies were surrounded by a thin cell wall (Figure 8C) and embedded in a somewhat multi-layered sheath with varying electron densities from electron dense (Figure 8D) to loose and apparently disintegrating (Figure 8F, arrow).

#### Habitat

Hypolithic biofilm under quartz stones.

#### Etymology

‘*diffluens*’ – ‘diffluent’, because of the diffluent gelatinous sheath of older colonies that release single unsheathed cells.

#### Type Locality

CHILE – Coastal Range of the Atacama Desert, Pan de Azúcar National Park, 25°58′24″S; 70°36′56″W, 667 m a.s.l., collected (18.07.2017) and isolated by Patrick Jung.

#### Holotype

The preserved holotype specimen is available *via* the Herbarium Hamburgense, Hamburg, Germany (HBG-024928). 16S, ITS, and 23S rRNA gene sequence deposited as GenBank accession number MT986030. TEM samples embedded in resin are available for reference at the Department of Botany, University of Innsbruck.

#### Reference Strain

The reference strain *Gloeocapsopsis diffluens* sp. nov. PJ S16 is available at the culture collection DSMZ Braunschweig (DSM 109259).

#### Differentiation Against Other Species

*Gloeocapsopsis diffluens* sp. nov. differ from other *Gloeocapsopsis* species by having a uniform cell shape throughout all stages, and an uncolored sheath that becomes diffluent in older colonies releasing unsheathed single cells. Furthermore, older colonies form a *Coleochaete*-like growth pattern. Sheaths are not as layered as in *G. dulcis* when observed by TEM.

#### Phylogenetic Relations and Secondary Structure of the 16S–23S ITS

The sister clade of *Gloeocapsopsis* is *Gloeocapsa*. Based on 16S rRNA gene sequence analysis, *G. diffluens* sp. nov. PJ S16 is closely related to *G. dulcis* sp. nov. AAB1 (98.29%), *G. crepidinum* LEGE 06123 (98.15%), *Gloeocapsopsis* sp. CENA 327 (97.59%), and the unicellular thermophilic cyanobacterium tBTRCCn 23 (97.24%; accession number DQ471448). The p-distance of the 16-23S ITS region shows 20.7% dissimilarity with *G. dulcis* sp. nov. AAB1, and 19.9% with *G. crepidinum* LEGE 06123. The secondary structure of the main informative helices of the 16-23S ITS region (Figure 6) shows differences in paired regions and loops between *G. diffluens* sp. nov. PJ S16, *G. crepidinum*, and *G. dulcis* sp. nov. AAB1; a significant different structure of the D1-D1′ and the Box B region of *G. diffluens* sp. nov. PJ S16 compared to *G. dulcis* sp. nov. AAB1 and *G. crepidinum* LEGE 06123 was found based on several variable bases, insertions, and deletions resulting in additional loop structures.

## Discussion

### General Implications for the Taxonomy of Cyanobacteria

Kováčik et al. (2011) described that coccoid cyanobacteria are particularly heterogeneous and differing in important phenotypic and ultrastructural markers. The cyanobacterial systematics later proposed by Komárek et al. (2014) placed the coccoid cyanobacteria in five orders: Gloeobacterales, Synechococcales (both either lacking thylakoids or possessing parietal thylakoids), Pleurocapsales, Chroococcidiopsidales, and Chroococcales (characterized by complex thylakoid arrangements). The phylogenetic tree based on the 16S rRNA shown in Figure 2 reflects the nearly complete cyanobacterial systematics given in several recent articles with a focus on unicellular coccoid cyanobacteria (e.g., Wang et al., 2019). In our study, we show that the true Pleurocapsales and most of the Chroococcales tended to aggregate into a large cluster with Spirulinales and some polyphyletic genera like *Synechococcus* (assigned to Synechococcales).

However, the Oscillatoriales still show an undefined phylogeny, with some genera (*Cephalothrix*, *Aerosakkonema*, and *Microseira*) mixed into other cyanobacterial orders (Figure 2) and thus supporting the findings of Wang et al. (2019). The genus *Chroococcidiopsis*, herein referred to as ‘core Chroococcidiopsidales’, appears closely related to the Oscillatoriales genera *Cephalothrix*, *Aerosakkonema*, and *Microseira*, thus setting them apart from *Sinocapsa*, *Gloeocapsa*, *Aliterella*, *Gloeocapsopsis*, and *Chroogloeocystis*, which form a cohesive group herein called ‘out-group Chroococcidiopsidales’. This division in the Chroococidiopsidales, which is close to the monophyletic Nostocales, remains unresolved, along with the position of genera and strains close to *Chroococcidiopsis*, *Aliterella*, *Sinocapsa*, and *Gloeocapsopsis*.

The above addressed phylogenetic positions and taxonomically established genera and species are solely based on the 16S rRNA gene in the context of the polyphasic approach as the golden standard for cyanobacterial taxonomy. However, there are emerging attempts that allow much deeper insights by comparing whole genomes, such as, e.g., Herdman and Rippka (2017),^2^ the Genome Taxonomy Database^3^ or Mareš (2018) to which the phylogenetic situation presented here based on the 16S rRNA gene mostly fits. Some works on genome-based phylogenetics even go one step further by proposing new genus and species concepts (Walter et al., 2017; Salazar et al., 2020) without giving genus and species descriptions by ignoring morphological features of the investigated strains. Currently, these proposed taxonomic concepts have not gained acceptances among taxonomists working on cyanobacteria and, for example, the establishment of type strains solely on genome date has just recently been rejected by the International Committee on Systematics of Prokaryotes (the committee which governs the Prokaryotic Code; Sutcliffe et al., 2020; Hugenholtz et al., 2021).

Perplexed phylogenetic and taxonomic situations, such as those of the *Microcoleus steenstrupii* complex, have recently been tackled by sticking to the polyphasic approach (Salazar et al., 2020). This is not at last a comprehensive approach because genus and species descriptions are given but also type strains were assigned and cultures of the strains are publicly available, a scheme this work aims to tie on.

### Emendation of the Genus *Gloeocapsopsis*

This study includes the emendation of *G. crepidinum* with a full epitypification as a reference point for all other *Gloeocapsopsis* species. Although the genus *Gloeocapsopsis* Geitler ex Komárek was described first in 1925 (Geitler, 1925) and validated in 1993 (Komárek, 1993), it has gained little attention. This may be explained as its morphology can easily be confused with those of other common unicellular cyanobacteria, such as *Gloeocapsa* (from where the name *Gloeocapsopsis* comes from, meaning ‘looks like *Gloeocapsa*’) and *Chroococcidiopsis*. More than twenty years later, *Gloeocapsopsis* and *Gloeocapsa* were found to be closely related sister taxa (Komárek et al., 2014). The type species of the genus, *G. crepidinum*, was first described as *Protococcus crepidinum* by Thuret (1854) found at various coast-like environments and since then placed in five other genera. Finally, Ramos et al. (2010) isolated a morphologically similar strain first in 2006 and again in 2008 in a comparable environment that they thoroughly described. Based on these morphological and ecological similar – if not identical – features they proposed the isolate *G. crepidinum* LEGE 06123 as the new type species for the genus *Gloeocapsopsis* together with genetic information what was later on supported by Komárek et al. (2014) in the frame of the establishment of the currently valid classification system. Since then, nine species of *Gloeocapsopsis* were described (Guiry and Guiry, 2020), most of them solely based on morphology (Supplementary Tables 1 and 2). A common source of confusion is the fact that not only *Gloeocapsa* is a morphologically similar genus due to its unicellular organization in a common sheath (see basionyms of *Gloeocapsopsis* species in Supplementary Tables 1 and 2). Also, *Chroogloeocystis siderophila* (Brown et al., 2005) which is not an accepted entity is morphologically and phylogenetically related to *Gloeocapsa* and *Gloeocapsopsis*, but detailed data are missing. In addition, the package-forming members of the Pleurocapsales (e.g., *Pleurocapsa*), members of the Chroococcales (e.g., *Gloeothece*), or the closely related Chroococcidiopsidales (*Chroococcidiopsis*) often share ecological niches. Some *Gloeocapsopsis* species still reflected these species names (Supplementary Tables 1 and 2). Thus, chances are high that some species of the genus *Gloeocapsopsis* need to be revised after the analysis of their 16S rRNA gene sequences, which is the case of ‘*G. crepidinum*’ strains KM350251, KY129702, and KF498710. Based on the 16S rRNA, they are positioned outside the *Gloeocapsopsis* cluster, but close to the Pleurocapsacean genus *Foliisarcina* (see Figure 2) and thus need to be revised after a careful evaluation of their morphology.

The genus *Gloeocapsopsis* has been reported in a wide variety of habitats. There is the subaerophytic *G. aurea*, reported from deglaciated coastal regions (Mataloni and Komárek, 2004), the lithic species *G. chroococcoides* and *G. dvorakii* that are described from stones and walls in the Czech Republic (Hauer, 2007), and the two hypolithic species reported in this work.

The morphological characteristics of previously described species coincide with a number of other taxa (e.g., *G. chroococcoides* and *G. pleurocapsoides*) particularly the broad range in cell sizes. While most described species have a cell size around 4 μm in diameter, *G. magma* and *G. chroococcoides* have bigger cells of 8.8–15 μm (Supplementary Tables 1 and 2), questioning the present taxonomic status of it.

### *G. crepidinum*, *G. diffluens*, and *G. dulcis*

The two species here described showed a high 16S rRNA gene sequence similarity compared to *G. crepidinum* LEGE 06123 (98.77%, 98.15%) but were also closely related to each other (98.29%) and formed a distinct cluster close to other Chroococcidiopsidalean genera. A similar pattern is reflected once considered on genome level where *G. dulcis* AAB1 clusters in close vicinity to *Aliterella atlantica*, the only trustable Chroococcidiopsidalean references type strain (see footnote 3). In addition, the comparison of the secondary structures of the helices D1-D1′ and Box B of the 16-23S ITS region of these three species showed that while the structures of *G. dulcis* and *G. crepidinum* varied slightly, that of *G. diffluens* sp. nov. PJ S16 showed a significantly different folding with additional loops.

The two new species formally presented here were both isolated from hypolithic biofilms found under quartz stones in the Coastal Range of the Atacama Desert (Figure 1). Interestingly, they show a different morphology, with homogeneous cell sizes and a highly diffluent sheath in older colonies in the case of *G. diffluens* sp. nov., and two cell types in older colonies in the case of *G. dulcis* sp. nov (Figures 4, 5, 7, 8). Compared to other described species within the genus, both species have significantly smaller cell sizes and an uncolored sheath throughout their development (Supplementary Tables 1 and 2). TEM analyses of cells of both species confirm most of the characteristics observed by bright field microscopy given in the diagnoses (Figures 5, 8). The cells of both species were surrounded by a thin cell wall and embedded in a thick sheath composed of a layered extracellular polymeric substances (EPS), and a comparable EPS structure is found in *G. crepidinum* LEGE06123 (Ramos et al., 2010). Cells of all three species occurred in small colonies arranged in irregular packets. Cells were embedded in a sheath composed of EPS, as summarized by Pereira et al. (2013), which in the case of *G. diffluens* sp. nov. disintegrates at some point. In contrast, cells of *G. dulcis* sp. nov. were embedded in a thicker, lamellate sheath, mainly in packages of two and four cells. Sheath-less cells could only rarely be observed. Cells of both species actively divided under the conditions provided, as evidenced by the observation of many septa by TEM. Thylakoids showed a parietal arrangement (as expected for the group) with distinct phycobilisomes clearly visible, as well as carboxysomes and cyanophycin granules (Figures 5, 8), all in line with previous reports (e.g., Gonzalez-Esquer et al., 2016; Kurmayer et al., 2017). The positioning of the thylakoid membrane unveiled by TEM has been a crucial character for the definition of taxa for decades but recent advances of molecular approaches, and frequently applied TEM techniques showed that thylakoid arrangement has rather limited taxonomically informative apomorphics (Mareš et al., 2019). However, TEM images have a strong value in cyanobacterial taxonomy allowing an unbiased view on various morphological features, such as the quality of the EPS, formation of septae, or granulation providing addition information that light microscopy is not able to provide.

Interestingly, although *G. dulcis* sp. nov. and *G. diffluens* sp. nov. could be considered as ecotypes of a common *Gloeocapsopsis* population (as they both inhabit hypolithic biofilms in the Coastal Range of the Atacama), the analysis of their ITS gene regions showed that this is not the case. The p-distance analyses as a novel tool to discriminate between populations showed a difference greater than 15% between the three strains *G. crepidinum* LEGE 06123, *G. diffluens* sp. nov., and *G. dulcis* sp. nov., which exceeds the proposed cutoff level of 7% of discrimination between two species to be considered as different (González-Resendiz et al., 2019).

## Conclusion

Our report on the taxonomy of the coccoid cyanobacterial genus *Gloeocapsopsis* is the first to formally introduce new members of this genus using a polyphasic approach. The taxonomic placement of *G. diffluens* sp. nov. and *G. dulcis* sp. nov. in the genus *Gloeocapsopsis*, together with the epitypification of the type strain *G. crepidinum* strengthens the position of the ‘out-group Chroococcidiopsidales’ including the genera *Aliterella*, *Sinocapsa*, *Gloeocapsa*, *Gloeocapsopsis*, and *Chroogloeocystis*. This is in contrast to the situation of the genus *Chroococcidiopsis*, the ‘core Chroococcidiopsidales’, appearing to be split by a few Oscillatoriales genera. Thus, the emendation of the genus *Gloeocapsopsis* will help to further reclassify other morphologically similar isolates of this fascinating order of the phylum Cyanobacteria, such as *Chroogloeocystis siderophila*, and strains clustering outside of the genus *Gloeocapsopsis*.

## Data Availability Statement

The datasets presented in this study can be found in online repositories. The names of the repository/repositories and accession numbers can be found in the article/Supplementary Material.

## Author Contributions

PJ, BB, ML, and AA-B guided the work. PJ, AA-B, and CG-S isolated and sequenced the species. TM analyzed secondary structures. AH took transmission electron microscopy images and interpreted them while PJ wrote the manuscript that was edited by all other authors. All authors contributed to the article and approved the submitted version.

### Conflict of Interest

The authors declare that the research was conducted in the absence of any commercial or financial relationships that could be construed as a potential conflict of interest.

## Footnotes

^1^https://blast.ncbi.nlm.nih.gov/Blast.cgi

^2^http://cyanophylogeny.scienceontheweb.net/

^3^https://gtdb.ecogenomic.org/
