# Supplementary material for: Emendation of the Coccoid Cyanobacterial Genus Gloeocapsopsis and Description of the New Species Gloeocapsopsis diffluens sp. nov. and Gloeocapsopsis dulcis sp. nov. Isolated From the Coastal Range of the Atacama Desert (Chile)
Source: Front Microbiol. 2021 Jul 8;12:671742. doi: 10.3389/fmicb.2021.671742 (PMC8295473; doi:10.3389/fmicb.2021.671742)
Supplement: Supplementary file 1 [file Table_1.DOCX]

Supplementary Material

# Supplementary Data

**Supp. Tab. 1: Comparison of morphological features of described *Gloeocapsopsis* species.**

|  | ***G. diffluens*** | ***G. dulcis*** | ***G. ferruginea*** | ***G. crepidinum*** | ***G. pleurocapsoides*** | ***G. cyanea*** |
| --- | --- | --- | --- | --- | --- | --- |
| **Cell size** | 3.2x2.4 µm (4.0 µm diameter), spherical, more or less uniform | 2.4x2.2 µm (3.3 µm diameter), spherical, often angular clinched | (2)3-5(6.3) µm diameter, more or less spherical, irregularly rounded, | 4-8 µm diameter, (4.3-3.6 µm)* | (3.8) 5.1-11.5 µm diameter (4.8(8.8) 4.8-6.7 μm)*, spherical, hemispherical, quarter ball-shaped, angular-circular, elongated, tightly packed | 3.2-4.5 µm diameter, tetrads common, rounded to spherical |
| **Color** | Olive green to bluish | Olive green bluish to brownish | Pale greyish blue-green | Bluish* | Intensive blue-green | Dark blue |
| **Cell content** | Homogeneous, ungranulated, | Homogeneous | Finely and indistinctly granular |  | Homogeneus |  |
| **Sheath** | Colorless, unlamellated, limited, up to 1 µm | Colorless, slightly lamellated, limited, up to 1 µm | Firm, thick, distinct, smooth from outside, usually intensely yellowish-brown due to the inclusion of ferric precipitates | Firm, slim, unlamellated, yellow to brownish, 0.6 µm thick* | Clear, narrow, non-layered, cellular or rather wide, layered, yellowish to yellow | Slim, limited, mimicking cell form, slightly lamellated, colorless, |
| **Thylakoid** | Parietal | Parietal | - | Parietal* | - | - |
| **Colonies** | Rarely tetrad forming, up to 30 µm, rounded to flattened, hundreds of cells arrange in a *Coleochaete*-like, prenchymateous pattern where cells without sheaths are released at the periphery | Two types a) cells aggregated in tetrad-like packets with a firm sheath mimicking the shape of the cells; b) rounded colonies with a firm sheath, both up to 12 µm | Up to 20 µm | 12 µm* | Colonies microscopic, simple, rounded or elongated, up to 20 µm crosswise or complex irregular, up to 121 µm crosswise; colonies macroscopic, 12.7-74 µm* | Up to 57 µm |
| **Sheath** | Common, firm, limited, unlamellated, colorless; highly diffluent in older colonies | As above | As above | Colorless, diffluent at outer periphery, unlamellated | Up to 5 µm width, yellowish to intense yellow. (sometimes colorless), often thickened along the edge, dark brown to black, with an uneven, bumpy edge, sometimes a little layered | As above |
| **Habitat** | Hypolithic biofilm, Atacama Desert, Chile | Hypolithic biofilm, Atacama Desert, Chile | Epiphyllic, attached to filamentous algae, small lake NNW of Amai Dablang, 4650 m a.s.l. | Subaerophytic, lithic, on rocks, stones and wooden columns near coastlines; shallow intertidal puddles at Portugal* | Aerophytic to subaerophytic, epilithic on dry, temporarily moistened rocks, wet crust in the mossy turf, known from a number of mountainous regions of Europe, Asia, South America | Aerophitic, epilithic, Grotto, Greece, Carpathian biosphere reserve, Ukraine |
| **Reference** | This study | This study | Komárek and Watanabe 1998 | Translated from Geitler 1932; *after Ramos et al. 2010 | Translated from Kovalenko and Kislova 2009; *translated from Lastra and Seoane 2002 | Translated from Kovalenko and Kislova 2009 |

**Supp. Tab. 2: Comparison of morphological features of other described *Gloeocapsopsis* species.**

|  | ***G. polyedrica*** | ***G. aurea*** | ***G. magma*** | ***G. chroococoides*** | ***G. dvorakii*** |
| --- | --- | --- | --- | --- | --- |
| **Cell size** | 3.8-5.4 µm (5-9.2 µm)*, oval, hemispherical, sometimes angular, densely arranged | (3.2) 4–9 µm, spherical to irregular-spherical or semi-globose | 8.8-15 µm (3-7 µm)*, unregularly rounded to angular | 10-15(18) µm, irregularly spherical, oval or polygonal, | 6-10 µm (3.5-11.5 µm)*, irregularly spherical, oval or polygonal |
| **Color** | Blue-green | Pale blue-green or greyish | Yellowish, greenish to blue-green | Green |  |
| **Cell content** | - | Slightly granulated | - | - | Homogeneous, sometimes with several scarcely distributed granules* |
| **Sheath** | Grayish to dark grey, repeating the shape of the cells, clearer and darker at periphery | Sharply delimited, partly (in young colonies) colorless, later slightly lamellated, gold-yellow or rusty yellow-brown to orange, copying the outline of cells or cell clusters, never widened in the spherical envelopes | Up to 1.6 µm thick, firm, thin, limited, rust-colored, granulated, unlamellated | Closely follow the outline of the cell and are colorless or pale green to blackish, sometimes also slightly reddish to violet; sometimes granulated | Closely follow the outline of the cell and are colored in different intensities of orange-red, sometimes granulated. |
| **Thylakoid** | - | - | - | - | - |
| **Colonies** | Slightly rounded or flattened, up to 38 µm containing up to 12 cells | Irregular, packet-like, agglomerated, sarcinoid, microscopic or later forming thin macroscopic, granular, blackish mats or crusts | Rounded, unregularly, 21-140 µm but more often 100 µm, intensively rust colored to purple brown, | Spherical to irregular, often composed of subcolonies | Microscopic, spherical, gelatinous colonies up to 120 µm (140µm)* |
| **Sheath** | Inside colony grayish to dark grey, slightly or noticeably layered, along the edge of the colony clear, intensely dark purple, with a compacted, uneven, sometimes fringe edge | As above | As above | As above | As above |
| **Habitat** | Aerophytic, epilithic, Carpathian biosphere reserve, Croatia | Subaerophytic, growing as mats and crusts on stones, deglaciated, coastal regions of humid maritime Antarctica; culture approach was never successfull | Forms blackish (dry) to brownish (wet) crusts on rocks, rust-colored to purple brownish, never slimy, | Epilithic on rocks such as limestone and serpentinic rocks, Czech Republic | Epilithic limestone, or serpentine walls and outcrops, Czech Republic; xerotherm, serpentine, volcanic, Japan* |
| **Reference** | Translated from Kovalenko and Kislova 2009; *after Komárek and Anagnostidis 1999 | Mataloni and Komárek 2004 | Translated from Geitler 1932; *after Komárek and Anagnostidis 1986 | Hauer 2007 | Hauer 2007; *after Komárek and Anagnostidis 1986 |

**References:**

Geitler, L. (1930-32). Cyanophyceae. In: L. Rabenhorst, *Kryptogamenflora* 2e Aufl. 14: 1

Komárek, J., and Anagnostidis, K. (1986). Modern approach to the classification system of cyanophytes, 2 - Chroococcales. Arch. *Hydrobiol./Algolog*. Stud. 43: 157-226.

Komárek, J. (1993). Validation of the genera *Gloeocapsopsis* and *Asterocapsa* (Cyanoprokaryota) with regard to species from Japan, Mexico and Himalayas. Bulletin of the National Science Museum, Tokyo, *Series B (Botany)* 19(1): 19-37

Komárek, J., Anagnostidis, K. (1995). Nomenclatural novelties in chroococcalean cyanoprokaryotes. *Preslia*. Praha 67: 15-23.

Komárek, J., Watanabe, M. (1998). Contribution to the attached Cyanoprokaryotes from submerged biotopes in Sagarmatha National Park (Eastern Nepal). Bulletin of the National Science Museum *Series B (Botany)* 24(4): 117-135.

Kovalenk, О. В., Kislova, О. А. (2009). New and rare Cyanophyta-species (Cyanoprokaryota). *Algology* 19(2): 216-225.

Mataloni, G., Komárek, J. (2004). *Gloeocapsopsis aurea*, a new subaerophytic cyanobacterium from maritime Antarctica. *Polar Biol*, 27(10): 623-628.

Hauer, T. (2007). Rock-inhabiting cyanoprokaryota from South Bohemia (Czech Republic). Nova Hedwigia, 85(3-4): 379-392.
